# Improving Quality of Life in Bipolar Disorders with an Immersive Virtual Reality Remediation Training Randomized Controlled Trial (RCT)

**DOI:** 10.3390/jcm13133886

**Published:** 2024-07-02

**Authors:** Diego Primavera, Gian Mario Migliaccio, Valentino Garau, Germano Orrù, Alessandra Scano, Alessandra Perra, Samantha Pinna, Massimo Tusconi, Mauro Giovanni Carta, Federica Sancassiani

**Affiliations:** 1 Department of Medical Sciences and Public Health, University of Cagliari, Monserrato Blocco I (CA), 09042 Cagliari, Italy; alessandra.perra@unica.it (A.P.); samanthapinna1984@gmail.com (S.P.); maurogcarta@gmail.com (M.G.C.); federicasancassiani@yahoo.it (F.S.); 2 Department Human Sciences and Promotion of the Quality of Life, San Raffaele Open University, 00118 Rome, Italy; gianmario.migliaccio@uniroma5.it; 3 School of Dentistry, University of Cagliari, 09042 Cagliari, Italy; garauva@yahoo.it; 4 Department of Surgical Sciences, University of Cagliari, Cittadella Universitaria, Blocco I, Asse Didattico Medicina P2, Monserrato (CA), 09042 Cagliari, Italy; gerorru@gmail.com (G.O.); alessandrascano@libero.it (A.S.); 5 University Hospital of Cagliari, 09124 Cagliari, Italy; massimotusconi@yahoo.com

**Keywords:** advanced technology laboratory, bipolar disorder, rhythm dysregulation, virtual reality, mood disorder

## Abstract

**Background:** Health-related quality of life (H-QoL) is a critical measure in bipolar disorder (BD). Recent trials using virtual reality (VR) have shown potential in improving H-QoL. However, VR’s effect on the H-QoL of people with BD needs to be further explored. **Methods:** This study involved a secondary analysis of a feasibility randomized controlled trial, focusing on “quality of life”. Participants (aged 18–75) diagnosed with bipolar disorder were randomized into two groups. The experimental group used the CEREBRUM VR app, while the control group received the usual care. Quality of life was assessed using the Short-Form Health Survey (SF-12). **Results:** A total of 39 individuals in the experimental group and 25 in the control group represent the final samples. The results showed a greater improvement in the SF-12 total score in the experimental group (8.7%) compared to the control group (F = 66.851 *p* < 0.0001), specifically in the dimension of physical activity limitation, emotional impact, concentration, pain, calmness, energy levels, discouragement, and social activities. **Conclusions:** This study demonstrated an improvement in QoL for individuals with BD following a VR intervention. As a feasibility study, this secondary outcome needs to be confirmed by further phase III studies. If confirmed, VR could offer valuable rehabilitation tools and insights into the pathogenesis and treatment of BD.

## 1. Introduction

Bipolar disorder (BD) has a frequency of approximately 2–5% in the community [1,2]. The disorder represents a serious public health issue due to the significant impact on the social functioning of the individual and for consequences on the work, families, and social network of the person who suffers from it [2]. The course of bipolar disorder is characterized by acute episodes interspersed with long periods with few or no symptoms of depression or hypomania. Therefore, bipolar disorder can persist undiagnosed for a long period until the outbreak of a frank manic episode [3,4,5]. Bipolar disorder is diagnosed late also due to the stigma that accompanies it, which causes refusal of the diagnosis [3]. Furthermore, the first episode is often depressive [6], leading to the erroneous diagnosis of major depressive disorder [7,8]. In this frequent circumstance, the physician prescribes antidepressants, drugs that aggravate the long-term course and can induce the switch to mania in the short term [9]. Minor hypomanic episodes are often overlooked: not only is it difficult to differentiate them clinically from a normal period of a slightly euphoric mood but also the fact that the person goes through these periods after having sometimes suffered from depression make it even more difficult for the person to “finally feel happiness” accepting such a stigmatizing diagnosis. On average, the delay in diagnosis lasts approximately 10 years, and one-third of patients with bipolar disorder have been misdiagnosed and treated [10,11].

People with bipolar disorder have difficulty finding a job and maintaining stable emotional relationships [2]. The risk of suicide [12,13] and repeated hospitalization [13] is high. It can be said that bipolar disorder greatly compromises the quality of life of those who suffer from it. The construct of health-related quality of life (H-QoL) is complex, abstract, and multidimensional. As a result, different and perhaps contradictory definitions have been used in studies on H-QoL [14]. However, H-QoL encompasses more than the standard of living, including satisfaction with the living environment, physical and mental health, education, social competence, leisure time, and social belonging [14,15]. Subjective perceptions play a key role. The objective level of the individual economic and social ranking and the reference society are important to contextualize the results achieved, but the key is the subjective perception of a person’s well-being, which does not always perfectly match with the parameters of economic position and social recognition [14,15,16,17,18,19]. Within the framework of the modern concept of recovery [20,21,22], H-QoL is assumed as a measure of outcome of chronic diseases and is one of the more complex measures. In fact, considering that this construct summarizes other simpler outcome measures, H-QoL is even more relevant in illnesses and disorders with a higher impairment and impact on daily life [23]. Consequently, H-QoL has become an essential measure for evaluating the effectiveness of therapeutic and rehabilitation treatments. Given the current importance of the debate relating to the environment and well-being, the concept of H-QoL is now frequently used to compare the life satisfaction of populations in relation to the environment and pollution [24,25,26,27]. Obviously, even in studying the course and severity of bipolar disorder, the level of perceived health-related quality of life has become a fundamental measure [26,27,28]. A strong impairment of the quality of life is also detected in forms of bipolar disorder once classified as “sub-threshold” [2]; today, these forms tend to be considered as conditions worthy of attention (DYMERS), characterized by the severe impairment of the rhythms of social life and biorhythms and by the risk of evolving into frank bipolar disorders [28,29] or other mood or anxiety disorders [30]. This conception of mood disorders as a spectrum [31,32] is far from the current classifications adopted in psychiatry; however, it is in line with the so-called neo-Kraepelinian tradition [33,34,35]. It also underlines how individuals with the characteristics of hyperactivity and novelty seeking, who can reach a good level of social adaptation, are at risk, under stressful conditions, of developing the pre-morbid condition of DYMERS and then, if the stressful condition persists, a full bipolar disorder [36,37,38]. One of the indicators of this risk in people with hyperactivity and novelty seeking is just the compromise in terms of quality of life [2,39]. Recently, a feasibility trial (RCT, NCT05070065) applying virtual reality technologies to counteract the cognitive decline that often accompanies bipolar disorder [40] surprisingly highlighted, as a secondary result, that, at the end of the process, the people who had undergone an intervention with virtual reality presented an improvement in the perception of H-QoL, the level of which was higher than that of the control group, at the limits of statistical significance [41]. Given the relevance of the perception of quality of life in the course of bipolar disorder and its significance in the prevention of the at-risk condition, this secondary analysis aimed to verify the extent of the improvement achieved through exercise with virtual reality in the experimental group compared to the control group in relation to the general score on the scale used to measure health-related quality of life and also to the individual items. Since this was a phase II feasibility study, starting from the difference in the outcome between the experimental and control groups, the objective was also to calculate the sample size needed to conduct a phase III study that produces solid data on this outcome.

## 2. Methods

### 2.1. Study Design

This study involved a secondary analysis of a randomized controlled trial (NCT05070065, 2021, Clinical.Trial.gov https://clinicaltrials.gov/study/NCT05070065) [40,41] conducted in line with the CONSORT indications [42]. The present study dealt with the declared secondary outcome “quality of life” [40]. The previous RCT had a cross-over design, meaning the control group remained on a waiting list and subsequently received the treatment.

### 2.2. Sample

As fully explained in the already published article, which illustrated the results of the main outcomes in detail, the sample recruited was comprised of outpatients of the Center of Liaison Psychiatry and Psychosomatics of the “Ospedale Civile, San Giovanni di Dio” in Cagliari, Italy, with a clinical diagnosis of bipolar disorder according to the American Psychiatric Association DSM-IV criteria [43]. Participants were aged between 18 and 75, with no exclusion based on gender. People with a current manic or depressive episode were excluded due to the difficulty in adhering to a twice-weekly intervention and the significant bias introduced by including individuals in both euthymic and relapse phases. Additionally, individuals with severe eye diseases or epilepsy were excluded because the stimulation produced by virtual reality could cause the worsening or recurrence of these conditions. Informed consent was signed before the intervention. After the recruitment phase, eligible participants were randomized into two groups, with 25 in the experimental group and 25 in the control group. Due to the cross-over design, the experimental sample actually consisted of 50 individuals, while the control group was comprised of 25 individuals. The final sample, excluding dropouts, consisted of 39 individuals in the experimental group and 25 in the control group (no participants dropped out while on the waiting list).

The randomization of participants into two arms was conducted by a biostatistician using a computer software with an allocation ratio of 1:1. The biostatistician was blinded and did not participate in the recruitment, evaluation, or intervention phases.

### 2.3. Experimental and Control Interventions

CEREBRUM is an app for cognitive rehabilitation developed as immersive virtual reality software. It was implemented by a group of cognitive rehabilitation experts, clinicians, engineers, and IT technicians. The software is compatible with OculusGo, a CE-marked virtual reality headset. The software allows the person who uses it to face virtual situations and experiences that simulate everyday reality, structured in such a way that they can be used to strengthen the skills and resources that can facilitate dealing with the difficulties of current life, and it offers the vision of a virtual environment that immerses the person at 360°. The user exploring the scene must face problems and difficulties and answer the rehabilitator’s driver questions.

CEREBRUM allows a stricter interaction with the professional and a continuous monitoring compared to a traditional cognitive remediation intervention, without virtual reality. The software includes 52 exercises of varying difficulty and themes: 22 exercises focus on memory and learning, 10 focus on cognitive assessments, and 20 focus on attention and working memory. The different difficulty levels are, during the exercise, calibrated and selected by the professional to adapt to the specific resources and the functional needs of the user. The exercises must be neither too easy nor too difficult for that specific person.

The proposed program included 24 sessions of 45 min, with 2 sessions per week over 3 months. Participants complete all exercises by the end of the 24 sessions. Each exercise has three difficulty levels. Based on the cognitive assessment and level of functioning, participants begin with a level 1, 2, or 3 of difficulty. The greater the cognitive difficulty is, the lower the starting level will be. This approach ensures that the initial engagement is not too difficult for those with greater challenges and not too easy for those with higher cognitive performance.

The typical structure of each session was the following: welcome, initial light exercises on well-being, and orientation to the instrument and the new tasks that would be faced (5 min). This phase involved physical exercise education and an explanation of the importance of the specific cognitive skills that would be strengthened in the specific session and the importance of their acquisition for coping with the possible difficulties of daily life (in accordance with an evolutionary vision of bipolar disorder and social inclusion work). We then moved on to the actual exercise in VR with continuous feedback from the professional and post-exercise discussion. The exposure to virtual reality was supposed to last approximately 15 and no more than 20 min. Two exercises were therefore carried out in one session, interspersed with a break for discussion. The second exercise was conducted with the same method described above. A final discussion followed with the assignment of “homework”, i.e., practical suggestions on how to apply the skills acquired during a normal day.

The experimental intervention was manualized step by step. The entire path was designed to be human rights centered [44] and recovery oriented [45,46].

During the trial, the control group and the experimental group continued to receive the usual treatments (psychiatric care, pharmacotherapy, psychotherapy if required, and rehabilitation/social inclusion interventions). The control group, which received treatment as usual, remained on a waiting list and subsequently received the experimental intervention due to the cross-over design.

Therefore, the comparison of effectiveness was related to the experimental group treatment as usual (plus intervention with virtual reality) compared with the control group with treatment as usual (without intervention with virtual reality).

### 2.4. Instrument

The overall and each item score of the self-administered Italian version [47] of the Short-Form Health Survey (SF-12), a self-administered questionnaire comprising 12 items designed to evaluate two dimensions, physical health and mental health [48,49,50], was used as a measure of the perception of the H-QoL. It has demonstrated validity and reliability, with a Cronbach’s alpha of 0.69 [50]. Higher scores correspond to a better quality of life. Specifically, the items investigate: item 1, “In general, would you say your health is …”. (from excellent to poor); item 2, “Does your health now limit you in moderate activities such as moving a table, pushing a vacuum cleaner, playing bowls or going for a bike ride”; item 3. “Your health currently limits you from carrying out activities of moderate physical effort such as climbing a few flights of stairs?”; item 4, “In the last 4 weeks, have you experienced at work or in other daily activities, because of your physical health, did you perform less than you would have liked?”; item 5, “In the last 4 weeks, have you experienced at work or in other daily activities, because of your physical health, have you had to limit certain types of work or other activities?”; item 6, “In the last 4 weeks, because of your emotional state (such as feeling depressed or anxious), did you performed less than he would have liked?”; item 7, “In the last 4 weeks, because of your emotional state (such as feeling depressed or anxious), have you had a loss of concentration at work or other activities?”; item 8, “In the last 4 weeks, how much pain has hindered you in your usual activities (both at home and away from home)?”; item 9, “How long in the last 4 weeks did you feel calm and peaceful?”; item 10, “How much of the time in the last 4 weeks did you feel full of energy?”; item 11, “How much of the time in the last 4 weeks have you felt discouraged and sad?”; and item 12, “In the last 4 weeks, how long has your physical health or emotional state interfered in your social activities, family, friends?”.

### 2.5. Statistical Analysis

At T0, the distribution was assessed using the Kolmogorov–Smirnov normality test, while the homogeneity of variance was evaluated using Levene’s test. For variables that did not meet the assumptions of normality and homogeneity of variance, a non-parametric analysis was conducted using the Kruskal–Wallis test. For variables that were homogeneous, a parametric one-way ANOVA test was used to compare baseline (T0) homogeneity between the experimental and control groups.

The differences between T0 and T1 (by time) of the change in the experimental vs. control groups (by groups) were calculated by ANOVA one-way statistics with Bonferroni correction. The comparison for nominal variables was conducted by means of the chi-square with Yates correction. A *p* value < 0.05 was assumed as statistically significant. The sample size useful to conduct a phase III study that produces solid data on this outcome was calculated for alfa 0.05 and beta 0.2 and with a power of 0.89.

## 3. Results

Due to 11 (22%) dropouts from the experimental group, the final sample consisted of 39 individuals in the experimental group and 25 in the control group. As indicated in the previous study, the people who dropped out did not differ from those who remained in the study in terms of gender and age distributions. The two samples compared did not differ in terms of age and gender of the members. Females predominated in both groups (Table 1). There were also no differences between the two groups in terms of educational level (Table 1). Moreover, the experimental and control groups did not differ in the variables analyzed in the secondary analysis, specifically the global score and individual items of the SF-12 (Table S1, Supplementary Material).

In Table 2, it can be seen that the SF-12 total score improved to a greater extent from T0 to T1 in the control group compared to the experimental group (2.67 ± 0.93 vs. 0.40 ± 1.29; F = 66.851, *p* < 0.0001) with 8.7% of gain in the experimental group (Table 2). Among the items of the SF-12, there was a statistically significant improvement in the control group in the items (see Table 2): item 3, “Your health currently limits you from carrying out activities of moderate physical effort such as climbing a few flights of stairs?” (0.23 ± 0.13 vs. −0.04 ± 0.14; F = 58.244, *p* < 0.0001), with 13.9% of gain in the experimental group; item 6, “In the last 4 weeks, because of your emotional state (such as feeling depressed or anxious), did you performed less than he would have liked?” (0.31 ± 0.10 vs. 0.08 ± 0.09; F = 86.988, *p* < 0.0001), with 19.6% of gain in the experimental group; item 7, “In the last 4 weeks, because of your emotional state (such as feeling depressed or anxious), have you had a loss of concentration at work or other activities?” (0.12 ± 0.09 vs. 0.04 ± 0.12; F = 9.252, *p* = 0.003), with 6.7% of gain in the experimental group; item 8, “In the last 4 weeks, how much pain has hindered you in your usual activities (both at home and away from home)?” (0.23 ± 0.20; 0.10 ± 0.22; F = 5.953, *p* = 0.018), with 3.9% of gain in the experimental group; item 9, “How long in the last 4 weeks did you feel calm and peaceful?” (0.63 ± 0.17 vs. −0.02 ± 0.22; F = 187.127, *p* < 0.0001), with 19.9% of gain in the experimental group; item 10, “How much of the time in the last 4 weeks did you feel full of energy?” (0.56 ± 0.18 vs. 0.40 ± 0.25; F = 8.853, *p* = 0.004), with 5.7% of gain in the experimental group; item 11, “How much of the time in the last 4 weeks have you felt discouraged and sad?” (0.48 ± 0.19 vs. 0.04 ± 0.24; F = 66.394, *p* < 0.0001), with 15.6% of gain in the experimental group; and item 12, “In the last 4 weeks, how long has your physical health or emotional state interfered in your social activities, family, friends?” (0.28 ± 0.19 vs. 0.12 ± 0.26; F = 8.076, *p* = 0.006), with 4.7% of gain in the experimental group.

The scores of the other items did not present statistically significant differences in gain between the two groups; in none of the 12 items was there a significant improvement in the score of the control group (Table 3).

## 4. Discussion

The study suggests that a cognitive remediation intervention administered in a full virtual reality environment modified the perception of the health-related quality of life after three months in the experimental group of people with bipolar disorders to a greater extent than in the control group. In addition to the total score of the SF-12 questionnaire, the improvement was greater in the control group also in 8 out of the 12 items of the instrument, with a clearly perceptible difference in item 3 (“Your health currently limits you from carrying out activities of moderate physical effort such as climbing a few flights of stairs”), item 6 (“In the last 4 weeks, **because of your emotional state (such as feeling depressed or anxious), did you perform less than you would have liked?”), item 9 (“How long in the last 4 weeks did you feel calm and peaceful?”), and item 11 (“How much of the time in the last 4 weeks have you felt discouraged and sad?”). In essence, the general feelings of well-being and greater confidence appeared more radically modified. Taking into account that there was a loss of 22% in the experimental sample alone (14.7% in the total sample), the study also demonstrated that it is necessary to conduct a phase III study on approximately 200 people, so that the result of this outcome would be sufficiently reliable (i.e., alpha 0.05, beta 0.2, study power 0.8) to confirm the differences in the total score SF-12 and in the major items where there was a significant improvement in the experimental group.

The study obtained an exceptional and unexpected result considering the relevance of the perception of health-related quality of life in bipolar disorders as a measure of the state of the pathology [51,52,53], as a tool for measuring the course [54,55,56], as a warning of subsequent crises in people without or with sub-threshold symptoms [28,39,57,58,59], and, above all, as a complex indicator of general well-being because it is a synthesis of simpler indicators.

First, it must be underlined that virtual reality has been used together with brain imaging techniques in bipolar disorder in fundamental studies to analyze the characteristic response to social competence tasks [60,61,62,63,64,65]; other effectiveness studies are non-existent. It should therefore be specified at the outset that the apparently greater result obtained with the use of virtual reality reflects some characteristics of the tool compared to classical techniques when applied in bipolar disorder. A first element may be linked to the intrinsic properties of virtual reality, which offers very stimulating and fun exercises; it is therefore not surprising that the method may be more incisive in people with novelty-seeking characteristics such as those with a bipolar temperament [66,67]. These same people could find it more boring to work in front of a computer in a fixed location according to a classic model of cognitive rehabilitation. However, it must be taken into account that this path was initially conceived only to counteract cognitive decline; therefore, the characteristics of “novelty-seeking acceptable” probably overlap with the fact that the method of administration, with gradual exercises, constant interaction with the supervisor, in a virtual environment where it is easier to continuously measure oneself without fear of external judgments, is probably able to affect those cognitive aspects of reward prediction that are inefficient in bipolar disorder [68,69,70].

It is known that people with bipolar disorder, even in euthymia, show difficulties in social cognition skills [59,60]. Social cognition involves both cognitive and affective skills, deficits of which have been linked to mentalizing disorders in bipolar disorders [61,62,63], i.e., difficulty understanding one’s own and others’ emotions and intentions [62,63]. With the combined use of virtual reality and imaging, it was discovered that when faced with emotional conditions that involve complex responses, people with bipolar disorder (unlike controls) reduce activations in the “mirror neuron system” (right inferior frontal cortex, insula, and premotor cortex) [61]. It is therefore an incorrect way (at least in some specific circumstances) of using cognitive processes to bridge circumstances that would require a combined analysis of emotions and cognitive responses. The fact that this particular path can impact these processes could justify an improvement in such a complex outcome as the perception of H-QoL, where items related to self-esteem and feeling confident and calm improved the most. This could be an interpretation, particularly regarding the improvement of items related to social and emotional aspects (items 6, 8, 9, 11, 12), which are fundamental in bipolar disorder, as previously highlighted in the rationale.

### 4.1. Limitations

As this was a phase II feasibility study in which quality of life was a secondary outcome, it is urgent that this study be replicated with a sufficient sample size in phase III. Another limitation is the small sample size, coherent with a phase II study.

### 4.2. Implications for Research

Trying to understand why a cognitive rehabilitation intervention can achieve such an unexpected improvement in such a complex and multifaceted outcome measure can open more general fields of interest on an etio-psychopathological level. If confirmed, these results in phase III could not only provide an excellent tool for rehabilitation in bipolar disorder but also offer valuable insights into the pathogenesis of the disorder.

## 5. Conclusions

This study showed an improvement in the quality of life in a sample of people with bipolar disorder following the application of a full virtual reality exercise designed to counteract cognitive deficits.

Specifically, the improvements in quality of life suggest that virtual reality-based interventions may effectively target cognitive and emotional dysregulation in bipolar disorder. This could lead to a better understanding of the underlying mechanisms and contribute to the development of more targeted and effective therapeutic strategies.

Furthermore, the findings highlight the potential of virtual reality as a novel and engaging therapeutic modality, which could be particularly beneficial for individuals with bipolar disorder who exhibit novelty-seeking traits and may find traditional cognitive rehabilitation approaches less appealing. Overall, the study underscores the importance of innovative approaches in the treatment of complex psychiatric conditions and the need for further research to validate and extend these promising findings.

## Figures and Tables

**Table 1:** Experimental and control samples at baseline (T0).

	Experimental Sample	Control Sample	Differences
Female	64.1%	72%	Chi-square 1 df = 0.431; *p* = 0.512
Age (mean ± standard deviation)	47.51 ± 13.52	46.28 ± 13.40	F 1.63 df = 0.127; *p* = 0.723
Educational level: >Middle School (Degree, High school) <Middle School (Middle school, Primary school)	25 14	16 9	Chi-square 1 df = 0.001; *p* = 0.993

**Table 2:** Difference by time (T0–T1) and groups (experimental vs. control) of the total score of SF-12 and the score of each item of SF-12.

	T0 (Mean ± SD)	T1 (Mean ± SD)	Differences (Mean ± SD)	ANOVA One-Way 1.63 df	Gain (%)
**Global** (*N* = 39) EX	25.95 ± 8.44	28.62 ± 9.193	2.67 ± 0.93	F = 66.851 *p* < 0.0001	8.7
**Global** (*N* = 25) CO*N*	28.08 ± 7.129	28.48 ± 8.842	0.40 ± 1.29		
**ITEM 1** (*N* = 39) EX	2.33 ± 0.94	2.48 ± 0.95	0.15 ± 0.24	F = 0.033 *p* = 0.856	0
**ITEM 1** (*N* = 25) CO*N*	2.56 ± 0.80	2.72 ± 1.04	0.16 ± 0.14		
**ITEM 2** (*N* = 39) EX	1.84 ± 0.83	1.89 ± 0.84	0.05 ± 0.14	F = 0.662 *p* = 0.419	0
**ITEM 2** (*N* = 25) CO*N*	2.04 ± 0.77	2.12 ± 0.81	0.08 ± 0.15		
**ITEM 3** (*N* = 39) EX	1.94 ± 0.84	2.17 ± 0.78	0.23 ± 0.13	F = 58.244 *p* < 0.0001	13.9
**ITEM 3** (*N* = 25) CO*N*	2.16 ± 0.73	2.12 ± 0.58	−0.04 ± 0.15		
**ITEM 4** (*N* = 39) EX	1.36 ± 0.48	1.38 ± 0.49	0.02 ± 0.03	F = 2.156 *p* = 0.147	0
**ITEM 4** (*N* = 25) CO*N*	1.44 ± 0.49	1.43 ± 0.49	0.01 ± 0.02		
**ITEM 5** (*N* = 39) EX	1.38 ± 0.48	1.48 ± 0.49	0.10 ± 0.08	F = 0.863 *p* = 0.356	0
**ITEM 5** (*N* = 25) CO*N*	1.36 ± 0.48	1.44 ± 0.49	0.08 ± 0.09		
**ITEM 6** (*N* = 39) EX	1.17 ± 0.38	1.48 ± 0.49	0.31 ± 0.10	F = 86.988 *p* < 0.0001	19.6
**ITEM 6** (*N* = 25) CO*N*	1.28 ± 0.44	1.36 ± 0.48	0.08 ± 0.09		
**ITEM 7** (*N* = 39) EX	1.20 ± 0.40	1.38 ± 0.48	0.12 ± 0.09	F = 9.252 *p* = 0.003	6.7
**ITEM 7** (*N* = 25) CO*N*	1.32 ± 0.46	1.36 ± 0.48	0.04 ± 0.12		
**ITEM 8** (*N* = 39) EX	3.30 ± 1.41	3.53 ± 1.29	0.23 ± 0.20	F = 5.953 *p* = 0.018	3.9
**ITEM 8** (*N* = 25) CO*N*	3.34 ± 1.35	3.44 ± 1.52	0.10 ± 0.22		
**ITEM 9** (*N* = 39) EX	2.97 ± 1.18	3.64 ± 1.31	0.63 ± 0.17	F = 187.127 *p* < 0.0001	19.9
**ITEM 9** (*N* = 25) CO*N*	3.32 ± 1.16	3.28 ± 1.45	−0.04 ± 0.22		
**ITEM 10** (*N* = 39) EX	2.79 ± 1.11	3.35 ± 1.31	0.56 ± 0.18	F = 8.853 *p* = 0.004	5.7
**ITEM 10** (*N* = 25) CO*N*	2.92 ± 1.32	3.32 ± 1.15	0.40 ± 0.25		
**ITEM 11** (*N* = 39) EX	2.82 ± 1.37	3.30 ± 1.24	0.48 ± 0.19	F = 66.394 *p* < 0.0001	15.6
**ITEM 11** (*N* = 25) CO*N*	3.08 ± 1.05	3.12 ± 1.45	0.04 ± 0.24		
**ITEM 12** (*N* = 39) EX	2.97 ± 1.27	3.25 ± 1.10	0.28 ± 0.19	F = 8.076 *p* = 0.006	4.7
**ITEM 12** (*N* = 25) CO*N*	2.96 ± 1.11	3.08 ± 1.41	0.12 ± 0.26		

**Table 3:** Sample size calculator for a phase III study.

	Experimental Sample Gain % vs. Control Sample	Sample Required for Alpha 0.05, Beta 0.2, Power 0.8
SF overall size	8.7	146 (73 Ex vs. 73 Control)
SF-12 **ITEM 3**	13.9	102 (51 Ex vs. 51 Control)
SF-12 **ITEM 6**	19.6	70 (35 Ex vs. 35 Control)
SF-12 **ITEM 7**	6.7	224 (112 Ex vs. 112 Control)
SF-12 **ITEM 8**	3.9	392 (196 Ex vs. 196 Control)
SF-12 **ITEM 9**	19.9	68 (34 Ex vs. 34 Control)
SF-12 **ITEM 10**	5.7	266 (133 Ex vs. 133 Control)
SF-12 **ITEM 11**	15.6	90 (45 Ex vs. 45 Control)
SF-12 **ITEM 12**	4.7	324 (162 Ex vs. 162 Control)

## Data Availability

Data are contained within the article.

## Supplementary Materials

The following supporting information can be downloaded at: https://www.mdpi.com/article/10.3390/jcm13133886/s1, Table S1. Normality test in the Experimental group (EX) and Control group (CON), homogeneity of variance between EX and CON, parametric and no parametric test beteìween EX and CON at baseline (T0).
