# Supplementary material for: Improving Quality of Life in Bipolar Disorders with an Immersive Virtual Reality Remediation Training Randomized Controlled Trial (RCT)
Source: J Clin Med. 2024 Jul 2;13(13):3886. doi: 10.3390/jcm13133886 (PMC11242424; doi:10.3390/jcm13133886)
Supplement: Supplementary file 1 [file jcm-13-03886-s001.zip › jcm-3071624-supplementary.pdf]

S1. Normality test in the Experimental group (EX) and Control group (CON), homogeneity of variance between EX and CON, parametric and no parametric test beteìween EX and CON at baseline (T0)

| | Normality test (p) | EX vs CON Homogeneity of Variance (F, p) | EX vs CON No Parametric test (H, p) | EX vs CON Parametric test (F, p) |
|---|---|---|---|---|
| **Global** (N=39) EX | p=0.855 | F=0.924 | | F=1.023 |
| **Global** (N=25) CON | p=0.685 | p=0.340 | | p=0.316 |
| **ITEM 1** (N=39) EX | p=0.000 | F=0.021 | H=1.726 | F=1.020 |
| **ITEM 1** (N=25) CON | p=0.101 | p=0.888 | p=0.188 | p=0.317 |
| **ITEM 2** (N=39) EX | p=0.003 | F=1.261 | H=0.8 | F=0.869 |
| **ITEM 2** (N=25) CON | p=0.179 | p=0.265 | p=0.371 | p=0.335 |
| **ITEM 3** (N=39) EX | p=0.010 | F=1.300 | H=0.875 | F=1.086 |
| **ITEM 3** (N=25) CON | p=0.107 | p=0.258 | p=0.349 | p=0.302 |
| **ITEM 4** (N=39) EX | p<0.000 | F=0.614 | H=0.138 | F=0.185 |
| **ITEM 4** (N=25) CON | p=0.001 | p=0.436 | p=0.710 | p=0.668 |
| **ITEM 5** (N=39) EX | p<0.000 | F=0.158 | H=0.027 | F=0.038 |
| **ITEM 5** (N=25) CON | p=0.002 | p=0.692 | p=0.868 | p=0.846 |
| **ITEM 6** (N=39) EX | p<0.000 | F=3.342 | H=0.454 | F=0.823 |
| **ITEM 6** (N=25) CON | p=0.000 | p=0.072 | p=0.500 | p=0.369 |
| **ITEM 7** (N=39) EX | p<0.000 | F=3.854 | H=0.593 | F=0.987 |
| **ITEM 7** (N=25) CON | p=0.000 | p=0.054 | p=0.440 | p=0.326 |
| **ITEM 8** (N=39) EX | p=0.164 | F=0.131 | | F=0.855 |
| **ITEM 8** (N=25) CON | p=0.088 | p=0.718 | | p=0.359 |
| **ITEM 9** (N=39) EX | p=0.022 | F=0.200 | H=1.257 | F=1.286 |
| **ITEM 9** (N=25) CON | p=0.195 | p=0.656 | p=0.262 | p=0.262 |
| **ITEM 10** (N=39) EX | p=0.109 | F=0.507 | | F=0.148 |
| **ITEM 10** (N=25) CON | p=0.254 | p=0.479 | | p=0.702 |
| **ITEM 11** (N=39) EX | p=0.107 | F=4.411 | H=0.552 | F=0.700 |
| **ITEM 11** (N=25) CON | p=0.024 | p=0.039 | p=0.457 | p=0.406 |
| **ITEM 12** (N=39) EX | p=0.124 | F=0.187 | | F=0.002 |
| **ITEM 12** (N=25) CON | p=0.081 | p=0.666 | | p=0.963 |
